# Verbal encouragement in coaching: enhancing small-sided game performance in youth basketball players

**DOI:** 10.3389/fpsyg.2025.1512803

**Published:** 2025-02-25

**Authors:** Osman Yılmaz, Haitham Jahrami, Ladislav Batalik, Khaled Trabelsi, Achraf Ammar, Yusuf Soylu

**Affiliations:** ^1^School of Physical Education and Sports, Osmaniye Korkut Ata University, Osmaniye, Türkiye; ^2^Department of Psychiatry, Ministry of Health, Manama, Bahrain; ^3^Department of Psychiatry, College of Medicine and Medical Sciences, Arabian Gulf University, Manama, Bahrain; ^4^Department of Rehabilitation, University Hospital Brno, Brno, Czechia; ^5^Department of Physiotherapy and Rehabilitation, Faculty of Medicine, Masaryk University, Brno, Czechia; ^6^Department of Rehabilitation, Faculty of Medicine, Masaryk University, Brno, Czechia; ^7^Department of Public Health, Faculty of Medicine, Masaryk University, Brno, Czechia; ^8^Department of Movement Sciences and Sports Training, School of Sport Science, The University of Jordan, Amman, Jordan; ^9^Research Laboratory: Education, Motricity, Sport and Health, EM2S, LR19JS01, University of Sfax, Sfax, Tunisia; ^10^High Institute of Sport and Physical Education of Sfax, University of Sfax, Sfax, Tunisia; ^11^Department of Training and Movement Science, Institute of Sport Science, Johannes Gutenberg-University Mainz, Mainz, Germany; ^12^Research Laboratory, Molecular Bases of Human Pathology, LR19ES13, Sfax, Tunisia; ^13^Faculty of Sports Sciences, Tokat Gaziosmanpasa University, Tokat, Türkiye

**Keywords:** coach, coach encouragement, verbal feedback, game performance, technical performance, verbal encouragement

## Abstract

**Background:**

Verbal coach encouragement is a key motivational strategy that enhances skill development, game strategy execution, and team cohesion. In youth basketball, where players are still developing technical and decision-making abilities, coach encouragement can play a crucial role in sustaining effort, improving focus, and fostering resilience under small-sided games (SSGs). This study investigated how coach encouragement (CE) influences young basketball players’ technical performance and psychophysiological responses during competitive gameplay.

**Methods:**

Sixteen male basketball players (age = 15.19 ± 1.05 years) voluntarily participated in the study. Heart rate, ratings of perceived exertion, mood states and technical activities were recorded with coach encouragement and without coach encouragement during SSGs.

**Results:**

The results showed that SSGs with coach encouragement were associated with significantly higher heart rate (*p* ≤ 0.05, *d* = 1.69), maximum heart rate percentage (*p* ≤ 0.05, *d* = 1.15), ratings of perceived exertion (*p* ≤ 0.05, *d* = 1.69), enjoyment (*p* ≤ 0.007, *d* = 0.86), technical abilities (e.g., successful passes and shots; *p* ≤ 0.05, *d* ranging from 1.08 to 1.25), and fatigue (*p* ≤ 0.03, *d* = 1.47).

**Conclusion:**

The findings of this study highlight the importance of CE in improving the psychophysiological and technical abilities of young basketball players during SSGs. CE improves coach-athlete relationships and increases game performance.

## Introduction

Basketball is a court-based team game with varying intensities, qualities, unique game dynamics, and strategies (Stojanović et al., 2018). Recent systematic reviews have demonstrated that young players must develop exceptional aerobic fitness, anaerobic capacity, and technical skills to perform optimally during matches (Li et al., 2024). Therefore, coaches and practitioners emphasize game-based training to provide a natural environment in basketball and to enhance athletes’ physiological and technical skills (O’Grady et al., 2020). Contrary to traditional methods, such as running-based exercise, studies have indicated that game-based training practices, such as adjusting pitch size, number of players, rules, and tactics, simulate actual gameplay conditions and provide engaging, game-like experiences (Li et al., 2024; Sansone et al., 2020). Therefore, innovative training methods and interventions are explored to improve on-field performance between training sessions and games.

Previous studies have emphasized that various interventions used to improve basketball players’ performance include physical fitness (Russell et al., 2021), nutritional support (Tan et al., 2020), mental health treatments (Cao et al., 2022), and cognitive ability (Wolch et al., 2021). Furthermore, recent research has indicated that verbal encouragement (VE) effectively maintains player engagement during training sessions (Brandes and Elvers, 2017; Khayati et al., 2024; Sahli H. et al., 2022). Beyond physical and technical training, psychological interventions, such as verbal encouragement, play a key role in optimizing player engagement and performance (Khayati et al., 2024; Selmi et al., 2017; Selmi et al., 2023b; Soylu et al., 2024). Verbal feedback from coaches has been shown to enhance motor performance, mood, and motivation, particularly in high-intensity situations (Aydi et al., 2022; Engel et al., 2019; Hammami et al., 2023). These motivational strategies may help athletes sustain effort, improve focus, and build resilience in competitive environments.

Coaching behaviors and providing guidance and support are known to improve athletes’ physiological responses, time-motion characteristics (Brandes and Elvers, 2017), and game performance (Aydi et al., 2022) as external motivators. However, coaches often encourage and arouse players, aiming to enhance confidence, awareness, commitment, optimism (Wong, 2015), and athlete learning and performance (Corbett et al., 2024) while facing challenges and striving to reach full potential development. Most studies have indicated that VE from coaches can positively impact motor tasks (Hammami et al., 2023), physical skills, and mood change (Sahli F. et al., 2022), leading to quantifiable improvements in in-game performance (Soylu et al., 2024). Additionally, Engel et al. (2019) highlighted the role of verbal encouragement in enhancing player interactions and engagement during competitive gameplay. Rusmana et al. (2023) demonstrated that encouraging more tactical game intervention increased inside shooting, shooting, passing to the zone, and dribbling to the zone in male basketball players. Hammami et al. (2021) stated that VE positively affects repeated change-of-direction performance in basketball players. Another study revealed that basketball players’ game performance and internal load responses were unaffected by whether their coach gave them positive or negative verbal feedback (Mengi et al., 2023). In conclusion, coaching strategies such as VE can significantly affect player performance and psychophysiological responses to development and performance in young basketball players.

In basketball, the role of VE has primarily been studied in the context of physical fitness and cognitive ability improvement (Díaz-García et al., 2021; Hammami et al., 2023). However, research on the impact of competitive gameplay on game-specific technical skills and psychophysiological responses in youth basketball is limited. Although several studies have examined the impact of verbal encouragement (VE) on technical and psychophysiological responses in team sports such as soccer (Selmi et al., 2023b; Soylu et al., 2024) and handball (Sahli F. et al., 2023), limited research has specifically investigated its effects in basketball. Given the influence of VE on heart rate, perceived exertion, and technical performance in small-sided games (SSGs) (Mengi et al., 2023; Sánchez-Sánchez et al., 2018), further research is needed to validate these findings in basketball settings. This study aims to analyze the impact of VE on the performance of young basketball players during games. We hypothesize that verbal VE will enhance psychophysiological responses and technical actions during small-sided games.

## Methods

### Participants

Sixteen young male basketball players (age = 15.19 ± 1.05 years; height = 182.25 ± 5.98 cm; weight = 73.25 ± 6.86 kg) participated in the study. All players had at least 3 years of competitive basketball experience and trained an average of 4–6 sessions per week, each lasting 90–120 min. Participant inclusion criteria were: (i) under 17 years of male basketball players, (ii) each with more than 3 years of competitive game experience, (ii) no recent (last 6 months) injuries or illness, and (iii) participation in all experimental sessions. The exclusion criteria were as follows: (i) players who suffered any injury during the study, (ii) players who did not participate in the study on any day, and (iii) players who did not provide psychophysiological data. The study protocol and its potential implications, risks, and benefits were explained to participants and their parents. All participants were instructed to abstain from physical activity for 2 days and to abstain from eating for at least 3 hours before the games. This study followed the ethical principles outlined in the Declaration of Helsinki and was approved by the Research Ethics Committee (Protocol number: 07.02.2024/21222).

### Psychophysiological measurements

Psychophysiological measurements are widely used, cost-effective tools for evaluating internal load and player responses during training and competition (Polito et al., 2017). In this study, total distance covered was monitored using Polar V800 GPS watches with a 1 Hz frequency, a validated method for precision in team sports with a 3.2% margin of error (Gilgen-Ammann et al., 2020; Portas et al., 2010). While effective for straight-line movement, GPS accuracy declines during high-intensity activities (Aughey, 2011). Therefore, total distance was standardized per minute. Additionally, heart rate (HR) was recorded using a Polar H10 chest strap, and the average HR was used for analysis.

To assess players’ physiological and psychological responses to SSGs, two validated scales were used. The 20-point Borg Rating of Perceived Exertion (RPE) scale measured perceived exertion, capturing physical intensity and fatigue immediately after each SSG (Foster et al., 2021). The Borg scale is widely applied in sports science to quantify subjective training load. In contrast, the 24-item Brunel Mood Scale (BRUMS) assessed psychological states (Terry et al., 1999), including anger, confusion, depression, fatigue, tension, and vigor. The BRUMS, adapted for Turkish athletes (Soylu et al., 2023), was administered before and after each game to evaluate changes in emotional states. Participants responded to the question, “*How are you feeling right now?*,” using a scale from 0 (not at all) to 4 (extremely). This scale allowed for a detailed evaluation of how players’ mood states were influenced by the presence or absence of VE. By using both scales separately, the study was able to differentiate physical exertion (Borg RPE) from psychological mood responses, ensuring a comprehensive assessment of the impact of verbal encouragement on performance. To measure player enjoyment, the 8-item Exercise Enjoyment Scale, developed by Raedeke (2007), was administered. The study utilized the Turkish-adapted version validated by Soylu et al. (2023), which demonstrated strong psychometric properties in adolescent and adult athletes. Participants rated their enjoyment using a 1 to 7 Likert scale, with total scores ranging from 8 (lowest enjoyment) to 56 (highest enjoyment). To ensure responses captured immediate post-game experiences, Physical Enjoyment and Mood State assessments were conducted immediately after each SSG. The Physical Enjoyment Scale was completed within 5 min post-game, while Mood State was assessed both before and after each SSG to capture baseline emotional states and post-game affective responses. Finally, the RPE and enjoyment scores were recorded at the end of each game.

### Technical analysis

All SSGs were video-recorded using a Canon HF R806 high-definition camera (Canon, Tokyo, Japan) to ensure detailed performance analysis. The footage was analyzed using E-Analyze Digital Basketball Match Analysis Software (Espor Digital, Ankara, version 5.8), which allowed for an objective evaluation of game performance under both wVE and woVE conditions. The predefined coding system categorized technical actions into successful passes, dribbles, shots, defensive actions, and turnovers (Gok et al., 2023). Each action was rated using a binary success/failure classification to maintain objective assessment standards.

A successful pass was defined as one that reached the intended teammate without interception or deflection. Additional criteria considered accuracy (chest vs. bounce pass effectiveness), speed (pass strength), and timing (delivered under defensive pressure or in open space). A missed pass included any intercepted, deflected, or off-target passes that required significant adjustment by the receiver. A successful shot was any attempt that resulted in a made basket, while a missed shot included off-target, blocked, or rim-out attempts.

The analysis was conducted by an experienced basketball coach with over 3 years of coaching experience. While a structured coding framework was followed to maintain consistency, the evaluation was carried out by a single rater, which limits inter-rater reliability. The absence of a second independent evaluator is acknowledged as a limitation, as formal inter-rater reliability assessments could not be conducted.

Technical performance data were extracted and compared across conditions (wVE vs. woVE) to determine whether verbal encouragement influenced technical execution. The findings indicated that players performed significantly more successful passes and shots in the wVE condition, suggesting that coach encouragement positively impacted in-game decision-making and execution.

### Study design

**Figure 1:**
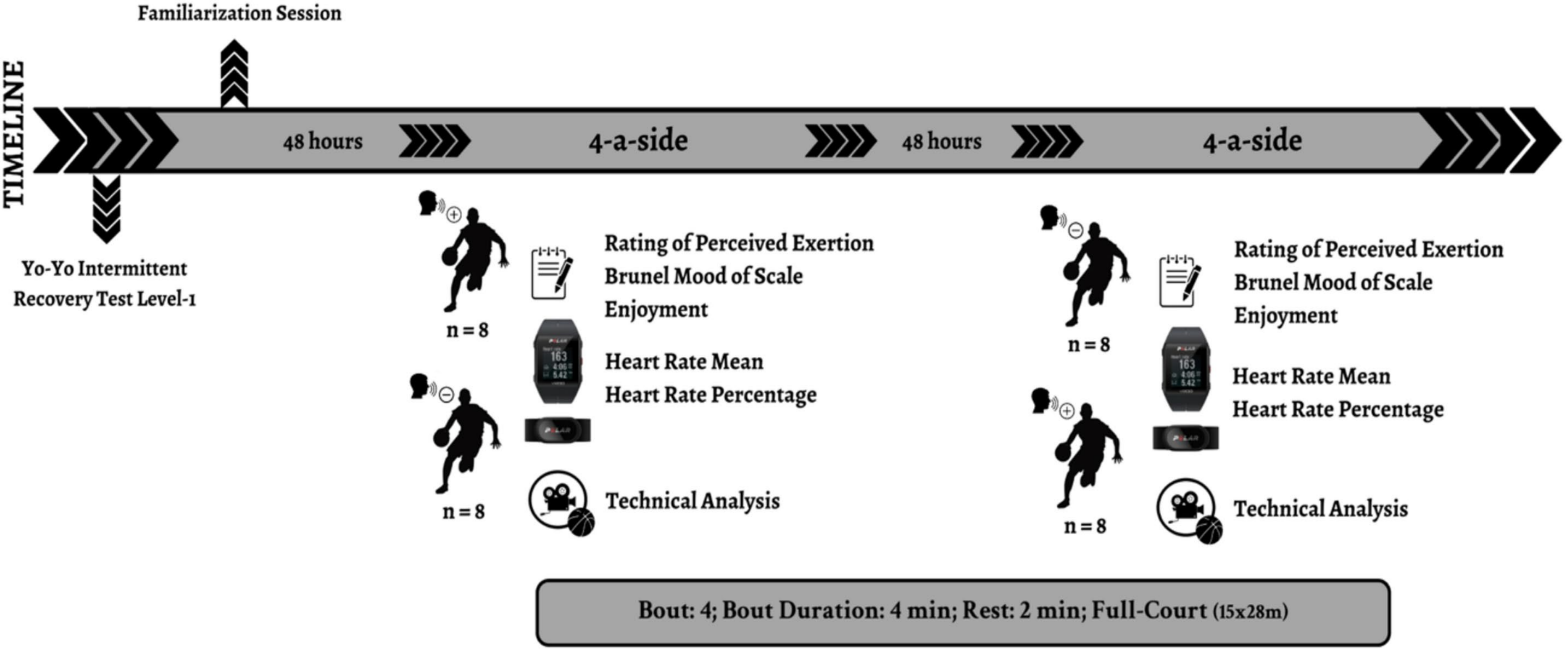
Study design.

The study followed a repeated-measures design in which participants played a series of SSGs under two conditions: with verbal encouragement (wVE) and without verbal encouragement (woVE). The study consisted of multiple phases, as illustrated in Figure 1.

The experiment began with a familiarization session, conducted 48 h before the first trial to minimize variability and ensure players were accustomed to the testing protocol. Before gameplay, participants underwent anthropometric measurements (height and body weight) and completed the Yo-Yo Intermittent Recovery Test (Yo-Yo IRT-1) to determine fitness levels and appropriately distribute players into balanced teams.

Each participant completed two experimental sessions, separated by at least 48 h to prevent fatigue accumulation. The SSGs were played in a 4 vs. 4 formats on a standard indoor basketball court, ensuring consistency across sessions. Each session lasted approximately 60 min, consisting of (i) A 10-min standardized warm-up, (ii) Four 6-min games, (iii) Three 3-min recovery intervals.

Throughout the study, psychophysiological responses were continuously monitored, including HR (beats·min^−1^), RPE, enjoyment, technical performance, and mood state profiles. Technical actions were recorded and analyzed using video-based performance analysis to compare execution across conditions (wVE vs. woVE).

The matches were designed to simulate real competitive gameplay scenarios, with teams maintaining their original composition across conditions to control for team dynamics. Additionally, all sessions were conducted at the same time of day (between 17:00 and 19:00) to mitigate potential circadian rhythm effects on performance outcomes (Pradhan et al., 2024).

VE included positive phrases, effort reinforcement, and technical feedback. According to previous research (Smith et al., 1977), coaches implement specific communication strategies using verbal behavior in wVE games. During the with wVE sessions, the coach provided structured VE throughout the games. The verbal encouragement included three main types: (1) positive reinforcement (e.g., “Great job!” or “Keep going!”), (2) effort reinforcement (e.g., “Push harder!” or “Stay focused!”), and (3) technical feedback (e.g., “Adjust your stance!” or “Look for open teammates!”) (Cushion et al., 2012). These phrases were delivered at regular intervals and were intended to enhance motivation, maintain intensity, and reinforce game strategies (Hammami et al., 2023; Soylu et al., 2024). In contrast, during the without coach encouragement (woVE) sessions, the coach remained silent and did not provide any verbal feedback.

### Statistical analysis

The sample size was estimated using G*Power software (version 3.1.9.4), which indicated that at least 16 participants were required, assuming an 80% power level (1-*β*), a significance level of 0.05 (*α*), and an effect size (d) of 0.8, based on previous studies on small-sided games (SSGs) (Khayati et al., 2024).

Data are presented as mean ± standard deviation (SD), 95% confidence interval (95% CI) of differences, and effect sizes (ESs). The normality assumption was validated using the Shapiro–Wilk test. HR, RPE, enjoyment, and technical activity were analyzed using a one-way variance analysis. The effect sizes (Cohen’s d) were also calculated for each dependent variable, including those classified as trivial (<0.2), small (0.2–0.6), moderate (0.6–1.2), large (1.2–2.0), very large (2.0–4.0), or extremely large (>4.0) (Hopkins et al., 2009). Mood states were analyzed using a two-way repeated measures ANOVA to examine the main effects of condition (wVE vs. woVE SSGs) and time, and their interaction effect (condition × time). When a significant interaction effect was observed, post-hoc analyses with Bonferroni corrections were conducted to identify specific differences between the conditions at each time point. The inter-individual variability in the psychophysiological and technical responses between the wVE and woVE SSGs was quantified using the coefficient of variation (CV%). Statistical analyses were performed using the SPSS software version 26.0 for Windows (SPSS Inc., Chicago, IL, USA). The significance level was set at *p* < 0.05. Mood state visualization was performed using the GraphPad Prism software.

## Results

Table 1 shows that the HR, %HRmax, RPE, and enjoyment values were significantly more significant in the wVE game than in the woVE game.

**Table 1:** Comparison of HR, RPE, and enjoyment scores between young basketball players in games wVE and woVE.

Variables	wVE Game	woVE Game	Mean difference	%95 CI	*p*	*d*	Descriptor
	X- ± SD	X- ± SD					
HR_(beat·min_^−1^_)_	181.92 ± 1.90^*^	177.84 ± 2.83	4.08	2.37–5.79	0.000	1.69	Large
%HR_(beat)_	92.20 ± 1.70^*^	90.14 ± 1.88	2.06	1.18–2.94	0.000	1.15	Moderate
RPE	14.39 ± 1.86^*^	10.22 ± 2.61	4.17	2.96–5.39	0.000	1.84	Large
Enjoyment	44.53 ± 3.94	41.47 ± 3.10	3.06	0.98–5.14	0.007	0.86	Moderate

HR, Heart Rate; %HR_max_, Percentage of maximum heart rate; RPE, Rating of perceived exertion; wVE, with verbal encouragement; woVE, without verbal encouragement.

Values are reported as means (X-) ± standard deviation (SD). Significant differences (*p* < 0.05) are marked with an asterisk (*); Effect size (Cohen’s d) interpretation: Small (0.2–0.6), Moderate (0.6–1.2), Large (>1.2).

Table 2 shows the technical responses of young basketball players in wVE and woVE games. There were significantly higher successful passes and shots in the wVE game than the woVE game.

**Table 2:** Comparison of technical performance variables between young basketball players in games wVE and woVE.

Variables	wVE Game	woVE Game	Mean difference	%95 CI	*p*	*d*	Descriptor
	X- ± SD	X- ± SD					
Successful pass	19.93 ± 1.84^*^	18.00 ± 1.15	1.93	1.06–2.82	0.000	1.26	Large
Unsuccessful pass	1.38 ± 0.81	1.50 ± 0.52	−0.12	−0.64–0.39	0.609	–	–
Interception	4.31 ± 0.95	4.69 ± 0.87	−0.38	−1.02–0.27	0.232	–	–
Lost Ball	1.69 ± 0.70	0.94 ± 0.44^*^	0.75	0.44–1.06	0.000	1.28	Large
Successful shot	5.31 ± 1.14^*^	4.06 ± 1.18	1.25	0.46–2.04	0.004	1.08	Moderate
Unsuccessful shot	4.56 ± 1.03	4.06 ± 0.85	0.5	−0.01–1.01	0.056	–	–

wVE: with verbal encouragement, woVE: without verbal encouragement. Values are reported as means (X̄) ± standard deviation (SD). Significant differences (*p* < 0.05) are marked with an asterisk (*); Effect size (Cohen’s d) interpretation: Small (0.2–0.6), Moderate (0.6–1.2), Large (>1.2).

The number of lost balls was significantly greater in the woVE game than that in the wVE game. There were no significant differences between unsuccessful passes, winning balls, or shots.

**Figure 2:**
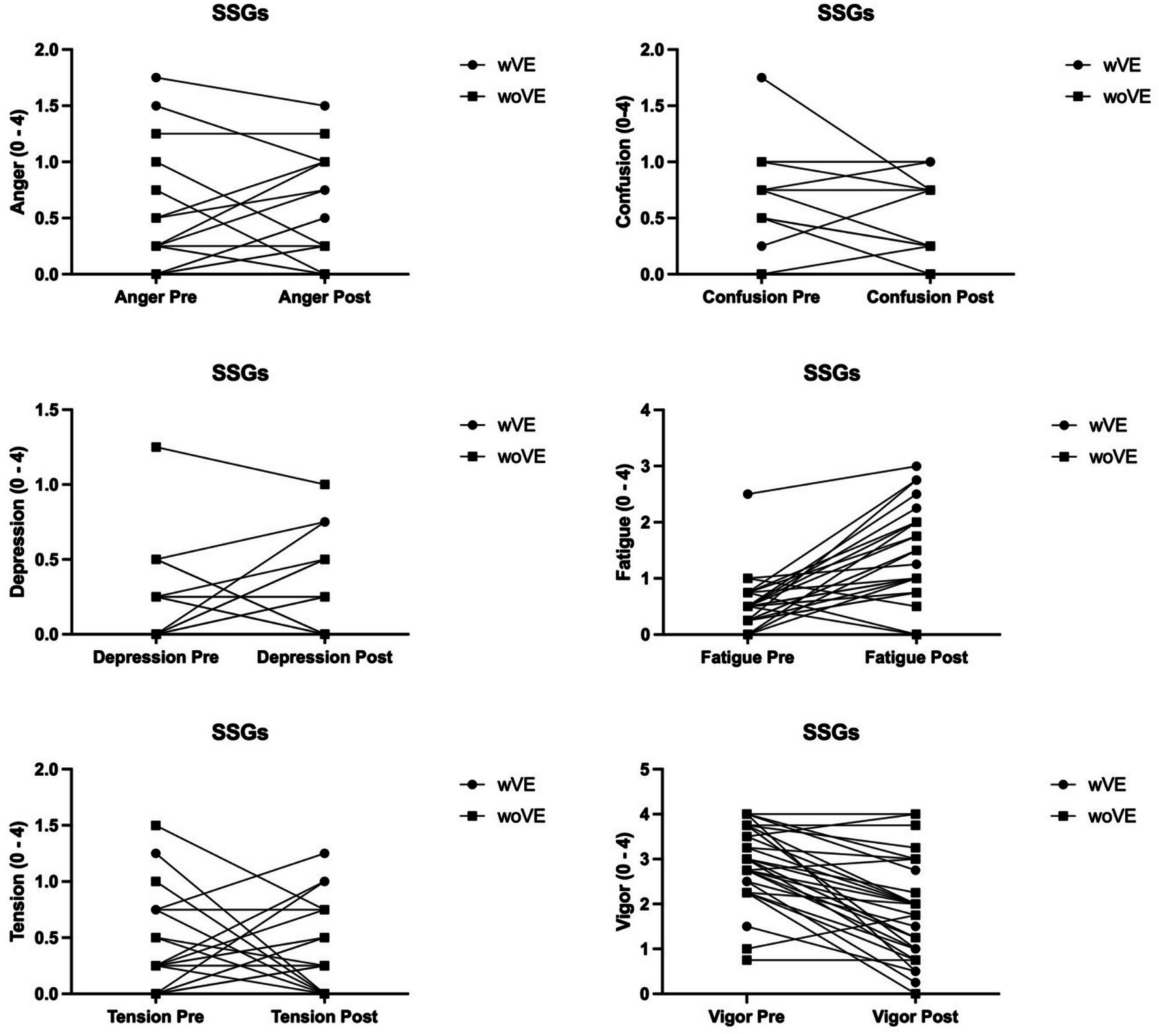
Mood responses of SSGs.

Figure 2 shows the mood responses of young basketball players in wVE and woVE games. Fatigue (*p* ≤ 0.03, *d* = 1.47) and vigor (*p* ≤ 0.00, *d* = −0.74) were significantly more significant in the wVE game than in the woVE game. There were no significant differences in anger (*p* ≤ 0.42), confusion (*p* ≤ 0.86), depression (*p* ≤ 0.76), or tension (*p* ≤ 0.41) between games.

## Discussion

This study examined the impact of coach encouragement (wVE) and no coach encouragement (woVE) on young basketball players’ gaming technical skills and psychophysiological responses. The study showed that the wVE game had a greater impact than the woVE game on multiple factors including HR (beats·min^−1^), %HRmax, RPE, enjoyment, technical skills (such as successful passes and shots), and fatigue levels. Lost ball and vigor states were significantly greater in the woVE game than in the wVE game. There were no significant differences in anger, confusion, tension-mood states, unsuccessful passes, unsuccessful shots, or technical abilities. The coaches monitored the intensity of the training load using HR and %HRmax. Additionally, RPE responses, frequently used by coaches and trainers, are fundamental, reliable, and cost-effective methods for assessing training load (Foster et al., 2021). Our study revealed that the wVE game had a more significant impact than the woVE game on HR, %HRmax, and RPE.

CE conveyed through words plays a crucial role in enhancing the performance of athletes in a wide range of sporting activities including basketball. This study showed that verbal CE increases physiological and perceptual factors such as HR and HRmax (%). Khayati et al. (2024) similarly revealed that coach-delivered VE revealed that increased enjoyment levels, positive mood state, physical activity intensity level and lower heart rate during half-court game SSGs. Sánchez-Sánchez et al. (2018) demonstrated that VE increased peak heart rate from sets 1 to 3 and elevated heart rate, perceived exertion, and offensive rebound performance in young female basketball players during a half-court 3′ side game. In addition to basketball, studies examining the effect of VE have shown that VE behaviors increase HR in males (Selmi et al., 2017; Selmi et al., 2023b; Selmi and Bouassida, 2017; Soylu et al., 2024) and female students soccer players (Hammami et al., 2023). In addition to game-based training, Midgley et al. (2018) reported that VE improves physiological measures, such as maximal heart rate, during maximal exercise testing. Unlike earlier research, Mengi et al. (2023) reported no significant differences in heart rate responses between no feedback and negative or positive feedback in basketball. The results indicate that verbal coaching can significantly impact an athlete’s heart rate response during basketball activities, specifically by increasing physiological arousal and effort (Mengi et al., 2023).

Our results showed that RPE increased verbal CE during small-sided basketball games. In agreement with our findings, Sánchez-Sánchez et al. (2018) indicated that 3-a-side game with verbal CE and dribbling showed greater RPE responses than a study without verbal CE. Similarly, In the study of Khayati et al. (2024) there were significant differences in RPE between the wVE and woVE groups only for session four, and there were no significant differences in the number of game factors for half-court basketball SSGs. These results align with earlier studies on soccer (Brandes and Elvers, 2017; Díaz-García et al., 2021; Sahli H. et al., 2020), which found that the use of CE led to an increase in RPE in the intensity of training sessions and enhanced soccer performance. In contrast to a previous study, Mengi et al. (2023) reported no substantial variations in RPE responses among the no-feedback, negative-feedback, and favorable-feedback conditions. Previous studies (Selmi et al., 2017; Selmi et al., 2023b) have shown that coaches’ positive encouragement during training significantly increases the training intensity. Soylu et al. (2024) noted that the influence of CE on game intensity may vary, depending on the nature and context of the feedback provided. Generally, constructive and encouraging feedback that enhances a player’s performance can increase the game intensity by fostering confidence and driving motivation (Grijalbo et al., 2022; Soylu et al., 2024). Given these results, coaches may benefit from VE by adjusting and optimizing the training intensity during games.

This study found that VE from coaches increased players’ perceived enjoyment even without other forms of encouragement. Previous small-sided basketball game studies with coach encouragement showed that VE did not change the perceived enjoyment levels (Khayati et al., 2024; Mengi et al., 2023). Although the results of existing basketball studies are contrary to our findings, it has been observed that players enjoy games more in CE practices in small-sided soccer games (Díaz-García et al., 2021; Sahli H. et al., 2020; Soylu et al., 2024). Bandura and Kavussanu (2018) found that athletes’ perceptions of authentic leadership positively influence their enjoyment and commitment. These relationships were mediated by perceived autonomy and trust, underscoring the role of supportive and empowering coaching in enhancing athletes’ enjoyment and commitment. Considering the outcomes of this study, the differences in wVE might be attributed to the traits of the players (students or trained players) and the aspects of the SSGs (number of bouts, bout duration, and pitch dimensions).

The current study demonstrated that technical activities including successful passes, and successful shot are more significant in the wVE game than in the woVE game. The players lost significantly more balls in the woVE game than in the wVE game. The encouragement provided by the coaches during the game can boost athletes’ motivation and encourage them to perform more successful passes and shots. However, encouragement can also pressure athletes and cause them to commit more ball losses. Similarly, a 12-week study of male basketball players revealed an increase in inside shooting, shooting, passing to the zone, and dribbling (Rusmana et al., 2023). Soylu et al. (2024) reported a significant increase in successful passes and shot technical performance in soccer players in 2-a-side, 3-a-side, and 4-a-side games played with small-sided games. In another study, VE significantly improved ball contact and successful technical performance among female soccer players (Hammami et al., 2023). Brandes and Elvers (2017) found that VE did not significantly affect the number of successful shots of a goal or the number of passes made in soccer.

Motivating, competitive, and challenging environments can help to increase athletes’ effort, focus, motivation, and enjoyment during training (Ekkekakis et al., 2011). Regarding the psychophysiological responses, the BRUMS data showed greater fatigue in the wVE game than in the woVE game. Encouraging feedback from coaches can motivate athletes and increase their effort to perform better. In addition, accurate and effective verbal feedback can optimize muscle performance in isokinetic testing, making the assessment and prevention of injury risk more reliable (Rendos et al., 2019). This highlights the importance of physiological and biomechanical considerations in optimizing performance and preventing injury. However, increased effort can also lead to additional pressure and fatigue among athletes, potentially explaining the significant fatigue observed in wVE.

Our findings are consistent with those reported by Soylu et al. (2024), who noted that tension, anger, and fatigue mood states were greater in wVE SSGs. In another study, Selmi and Bouassida (2017) revealed that tension increased while vigor decreased in woVE SSGs compared to wVE SSGs. Selmi et al. (2023a) reported that young soccer players experienced greater total mood disturbance, fatigue, tension, and anger and less vigorous mood states during repeated agility speed training in woVE than during wVE. Contrary to these results, another study showed that SSGs played with and without encouragement showed similar improvements in total mood disturbance and tension (Sahli H. et al., 2020).

Beyond the immediate psychophysiological and technical benefits of VE observed in this study, broader physical characteristics, such as body composition and movement behavior, may also influence performance and injury risk in youth basketball. Studies suggest that height, body mass index, and lower limb muscle power contribute to explosive movements, while factors like dynamic knee valgus increase knee injury risk (Lopes et al., 2018; Stojanović et al., 2018; Stojiljkovic et al., 2024). Given that VE enhances player engagement, effort, and movement intensity, it may also indirectly influence neuromuscular control and movement mechanics, which are critical for injury prevention. Future research should investigate how VE, combined with structured physical training, affects long-term biomechanical adaptations and injury risk mitigation in youth athletes.

The results of this study should be evaluated considering several limitations. Although the games were played considering the number of basketball team players, a larger sample size including a broader group of players is required to increase the generalizability of the results. The sample size of this study (*n* = 16) was determined using power analysis using statistical software ensuring adequate statistical power (80%) for detecting differences; however, the limited number of participants restricts the ability to generalize findings to a wider basketball population. Future studies should incorporate larger and more diverse samples to confirm these findings and improve external validity.

The participants were young basketball players with amateur experience. Therefore, the results may not be applicable to people of different ages, skill levels, or competition types. Only psychophysiological and technical responses to the wVE and woVE games were used in this study. The results may not wholly reflect the impact of physiological and kinematic factors. Additionally, technical action analysis was conducted by a single evaluator, which may introduce subjective bias as inter-rater reliability was not formally assessed.

The type of feedback provided during verbal encouragement (positive vs. corrective) may also influence player motivation and performance outcomes. While our study did not differentiate between feedback types, previous research suggests that positive reinforcement may enhance engagement and confidence, while corrective feedback could either improve skill acquisition or induce pressure depending on delivery style and context. Future studies should examine how different feedback types affect psychophysiological responses, technical performance, and long-term motivation in youth basketball players.

Finally, the study only assessed acute and short-term responses, which may not fully capture long-term effects. The current study also has limitations regarding an important determinant of the game’s time–motion characteristics, including high-intensity running activities, sprint counts, and covered distance.

## Conclusion

This study demonstrated that CE enhances psychophysiological responses and technical performance in young basketball players during games. wVE SSGs have greater HR, RPE, enjoyment, fatigue state, and successful passes and shots than woVE games. The state of vigor was greater in games without encouragement because of lower game intensity. VE significantly enhances athlete performance by increasing motivation, self-efficacy, and physical abilities. Coaches and support staff should provide positive verbal feedback to create a supportive and motivating environment that promotes optimal performance and well-being in basketball players.

## Data Availability

The raw data supporting the conclusions of this article will be made available by the authors, without undue reservation.
